# Making patient experience actionable: applying importance-performance analysis to guide improvements in Swedish healthcare

**DOI:** 10.3389/frhs.2026.1765906

**Published:** 2026-03-17

**Authors:** Therese Scott Duncan, Louise Lind, Li Åslund, Eva Dieker, Sara Riggare

**Affiliations:** 1 Participatory eHealth and Health Data Research Group, Department of Womens’ and Childrens’ Health, Uppsala University, Uppsala, Sweden; 2 Department of Psychology, Stockholm University, Stockholm, Sweden; 3 Mindler, Stockholm, Sweden; 4 Department of Clinical Neuroscience, Karolinska Institutet, Stockholm, Sweden

**Keywords:** importance-performance analysis, improvement science, learning health systems, patient experience, person-centered care

## Abstract

**Introduction:**

Understanding how well healthcare systems meet what patients value is central to person-centered care. As a first objective of this study, the alignment between what Swedish patients consider important in healthcare and what they experience in practice was examined, using an Importance–Performance Analysis (IPA) across eleven relational and functional care dimensions. A secondary objective was to assess how these patterns differ by disability level, an equity-relevant factor shaping healthcare needs and expectations.

**Methods:**

Anonymous survey data were collected from 1,036 adults across all Swedish regions who had accessed healthcare within the previous six months. Respondents rated the importance of, and experienced performance on, key care attributes including continuity, communication, shared decision making, timeliness, information access, and co-design. Mean importance and performance scores for each dimension were plotted in IPA grids for the full sample and for subgroups defined by self-reported disability due to long-term health issues (high, medium, none).

**Results:**

For the overall sample, most care dimensions fell into the “maintain performance” quadrant, indicating high importance and adequate performance, notably shared decision making, collaboration, communication preferences, responsiveness, and access to information. Relational continuity, informational continuity, support for self-care, and co-design were situated in the “lower priority” quadrant, reflecting lower relative importance and lower performance. For the total sample no dimensions appeared in the “focus efforts here” quadrant. Subgroup analyses revealed substantial inequities. Respondents with high levels of self-reported disability reported poorer health, greater difficulty accessing the knowledge they need, and lower performance ratings across all eleven dimensions. For this group, relational continuity and healthcare professionals' disease-specific knowledge shifted into the “focus efforts here” quadrant, indicating high importance coupled with significant performance gaps. These patterns were not observed among respondents with medium or no self-reported disability, for whom these dimensions were placed in areas indicating acceptable performance or lower priority.

**Discussion:**

The findings demonstrate that average patient-experience measures obscure meaningful disparities. IPA offers a practical decision-support method for identifying where improvement efforts should be targeted and for whom. Priorities for Swedish healthcare include strengthening continuity, enhancing informational support, and ensuring adequate professional knowledge for individuals with higher levels of self-reported disability. Used within iterative improvement frameworks, IPA can help move healthcare systems from measurement to meaningful, patient-informed change.

## 1 Introduction

Improvement science is an evolving field dedicated to strengthening the quality, safety, and responsiveness of healthcare systems. A core principle is that improvement efforts should be informed by what patients value and experience in practice (1). There is substantial evidence that both relational (interpersonal) and functional (organizational and technical) aspects of care are associated with clinical effectiveness, safety, and patient satisfaction (2). International frameworks reflect this alignment, both the World Health Organization's model for people-centered health services and the OECD Framework for People-Centered Health Systems emphasize voice, partnership, co-production, and the integration of patient preferences in care delivery (3, 4).

Learning Health Systems (LHS) further operationalize these principles by establishing continuous feedback loops in which patients, clinicians, and organizational leaders jointly identify priorities and use real-time data to drive improvement (5, 6). Within this context, patient engagement can be understood on two levels: *primary engagement*, which concerns involvement in one's own care (e.g., shared decision making, communication, self-management), and *secondary engagement*, which refers to participation in service design, research, and policy development (7–10). Both levels are essential for identifying gaps between what matters to patients and what healthcare systems deliver.

Existing literature shows that patients across diverse contexts value continuity of information, coordination between providers, shared decision making, timeliness, access, respectful communication, transparency, collaboration, and patient autonomy (11–14). Yet despite policy commitments to equity and person-centeredness, health systems often lack structured tools for assessing alignment between importance and performance from the patient perspective. This gap is especially relevant for people with chronic conditions or disabilities, who experience higher care needs and greater barriers to high-quality care (15, 16).

Importance–Performance Analysis (IPA) provides a practical way to address this assessment gap. Originally developed in marketing (17), IPA has also been applied in healthcare to visualize discrepancies between what users consider important and how well systems perform (18, 19). While IPA does not identify causal mechanisms, its quadrant structure offers actionable insights for quality improvement teams by highlighting areas where performance falls short of expectations, or where good performance should be maintained, see further descriptions under Materials and Methods below. The IPA method is particularly suited to improvement science because it can be embedded within Plan–Do–Study–Act (PDSA) cycles (20) and other iterative improvement methods.

Sweden's publicly funded healthcare system performs well across many clinical indicators (21), yet international comparisons show lower patient-reported satisfaction relative to other OECD countries (22) and notable disparities between patient groups (21). There are various surveys in use, for example the National Patient Survey (23) and the Quality from the Patient's Perspective survey (KUPP) (24). Both examples are used for evaluating healthcare settings and KUPP also examines patients’ perceptions of quality in healthcare. They do however not provide information on what patients consider important, which is one of the key aspects of IPA. To date, little is known about how well Swedish healthcare aligns with patient priorities across relational and functional domains, and to our knowledge, IPA has not previously been applied in this national context.

This study therefore firstly aimed to investigate the alignment between what Swedish patients consider important in healthcare and what they experience in practice, using an IPA across relational and functional care dimensions. A secondary aim was to explore how these patterns differ by level of disability, an equity-relevant factor that may shape both priorities and experiences. By identifying areas where performance diverges from patient-valued attributes, the study offers actionable insights for strengthening person-centered and equitable improvement efforts in Swedish healthcare.

## 2 Materials and methods

We conducted an observational, descriptive cross-sectional survey study. Data were collected using a study-specific online questionnaire, and the resulting data were analysed using Importance-Performance Analysis (IPA) to examine the alignment between patients’ ratings of importance and perceived performance across care dimensions.

### 2.1 Study and survey design

A study specific online survey was designed in Swedish including background information (demographics, attitudes on health-related information and knowledge, and self-rated health) and experiences and satisfaction from healthcare. Disability level was assessed using a two-step self-report item. Respondents were first asked whether they had a long-term health problem (yes/no). Those responding “yes” were then asked whether this problem reduced their ability to work or limited them in other daily activities (“yes, to a high degree”, “yes, to some degree”, “no, not at all”). We classified respondents as having high, medium, or no disability based on the reported degree of limitation. This measure reflects perceived activity limitation due to long-term health problems rather than clinically verified disability or diagnosis.

Our questions on healthcare experiences and satisfaction were formulated in Swedish based on inspiration from several sources, including the Picker Institute (25) and the Institute of Medicine (26), and the concept of continuity of care (27). In total, the survey included 11 dimensions of healthcare, on both relational and functional aspects of care (2) as well as codesign (28). Care dimensions covered were (see Table 1 for further explanations): relational continuity (Q1), healthcare professionals’ (HCP) level of knowledge related to individual patients’ concerns (Q2), shared decision making (Q3), collaboration with HCP (Q4), effective and timely care (Q5), mode of communication as preferred by patients (Q6), support for selfcare (Q7), HCP responsiveness to information from patient (Q8), informational continuity across settings (Q9), patients’ access to information (Q10), codesign of healthcare services (Q11). Respondents were asked to rate the importance of each of the care dimensions by responding to the statement: “It is important to me” [care dimension in Table 1] using a 5-point Likert scale (1 = strongly disagree, 3 = neutral, 5 = strongly agree). Data on performance were collected in a similar way by asking for responses to the statement: “My most recent healthcare visit fulfilled this for me” (for Q1-Q8) or: “healthcare fulfills this for me” (for Q9-Q11) on a 5-point Likert scale (1 = strongly disagree, 3 = neutral, 5 = strongly agree). For each care dimension there was also a non-mandatory free-text comment option. All questions, except free-text options, were mandatory to answer. The survey was pilot tested with three individuals, which resulted in minor changes to improve clarity and readability. See Supplementary Material for survey questions.

**Table 1:** List of care dimensions included in the survey.

No	Care dimension	Type of aspect
Q1	To meet the same HCP each visit (e.g., physician)	Relational
Q2	That HCP are knowledgeable about the disease(s)/problems I have	Functional
Q3	That HCP involve me in decisions regarding my care	Relational
Q4	To have good collaboration with HCP	Relational
Q5	To receive the right kind of healthcare without unnecessary waiting	Functional
Q6	To be able to communicate with healthcare in ways that are best for me—during visits, before visits, between visits, during investigations, and so on	Relational
Q7	That HCP support my selfcare, i.e., support me in taking care of my own health to the greatest extent possible	Relational
Q8	That HCP take onboard information I want to share.	Relational
Q9	That different HCP have access to the same information about me	Functional
Q10	To have access to the information that healthcare has about me (such as my medical record, test results, etc.)	Functional
Q11	To be able to participate in the development and organization of healthcare beyond my own visits, e.g., via patient advisory boards etc.	Relational

### 2.2 Data collection and analysis

Anonymous data were collected through an online survey from June to November 2024. The survey was distributed via patient organizations and networks, social media, as well as via an online healthcare provider (primary care) that advertised the survey through their application. To be eligible for the study, respondents should have had a healthcare visit within the last six months, be proficient in Swedish, and be over 18 years of age. A non-probability convenience sampling approach was chosen to maximize variability in respondent characteristics rather than statistical representativeness.

**Figure 1:**
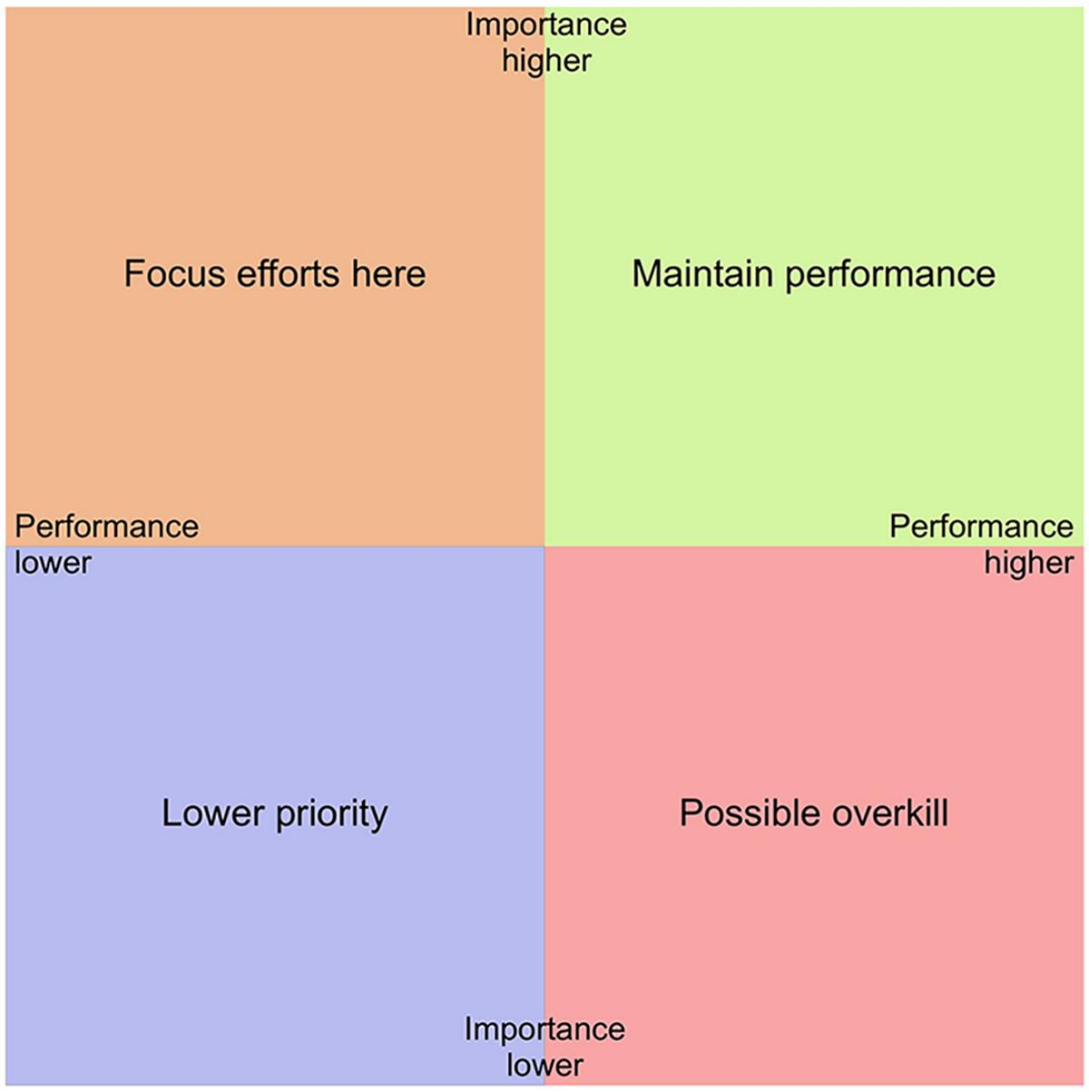
Example of an importance-performance grid with interpretations.

IPA is a diagnostic tool used to identify the strengths and weaknesses of a product, service, or system. It works by comparing how important different attributes are to users (importance) with how well those attributes are perceived to function (performance). These two dimensions are plotted on a grid divided into four quadrants, representing different relative levels of priority. By positioning each attribute in one of these quadrants, an IPA shows where performance aligns well with user priorities, and where gaps exist, thus highlighting areas that require attention, investment, or improvement (17). See Figure 1 for an example of an IPA grid with importance on the vertical axis, performance on the horizontal axis. Data from importance and performance ratings are ordinal and bounded, and can therefore be expected to be skewed and non-normally distributed. Since our aim is descriptive and comparative rather than inferential, mean values can often be used for IPA analysis also when data are not normally distributed (29).

Mean values were calculated for each of the questions Q1-Q11 and importance/performance respectively and plotted in an IPA grid. In addition, mean values were calculated for three levels of disability (high/medium/none) for our respondents. To show the level of spread in data, median values were also identified.

### 2.3 Ethics

Prior to initiating the study, an application regarding ethics was submitted to the Swedish Ethical Review Authority. Since data are collected anonymously, the authority determined that this study does not require ethical approval, as determined by the Swedish Ethical Review Authority (dnr: 2023-05537-01). Participants were given information about the study at the start of the survey and by submitting their responses, they gave their consent.

## 3 Results

### 3.1 Background

In total, 1,036 individuals responded to our survey, from all regions in Sweden (11% men, 48% aged 50–69). The number of responses from each region was approximately proportional to the national population distribution. Compared to the general population, our respondents consist of a larger proportion of people older than 50. When looking at our data per level of self-reported disability (hereafter disability), some important differences become apparent. In total, 30% of our respondents reported high levels of disability (GRP_A, *n* = 312), 37% reported medium (GRP_B, *n* = 379), and 33% reported no disability (GRP_C, *n* = 345), see Table 2 for background information.

**Table 2:** Background information per level of disability for survey respondents.

Level of disability	Gender			Age				Education level		
	Female	Male	Other	18–29	30–49	50–69	70-	Primary	Secondary	Tertiary
GRP_A	272	35	5	8	81	154	69	14	61	237
GRP_B	350	28	1	9	72	182	116	7	59	313
GRP_C	296	47	2	15	88	157	85	10	50	285
TOTAL	918	110	8	32	241	493	270	31	170	835
Occupation				Most recent healthcare encounter				Type of visit		
	Working	Retired (due to age)	On sick leave or disability	Other	Emergency room	Primary care	Secondary care	Other	Digital	In-person
GRP_A	24%	24%	44%	8%	7%	56%	32%	5%	22%	78%
GRP_B	47%	43%	4%	6%	3%	50%	42%	5%	26%	74%
GRP_C	61%	32%	1%	6%	5%	62%	28%	4%	36%	64%

**Figure 2:**
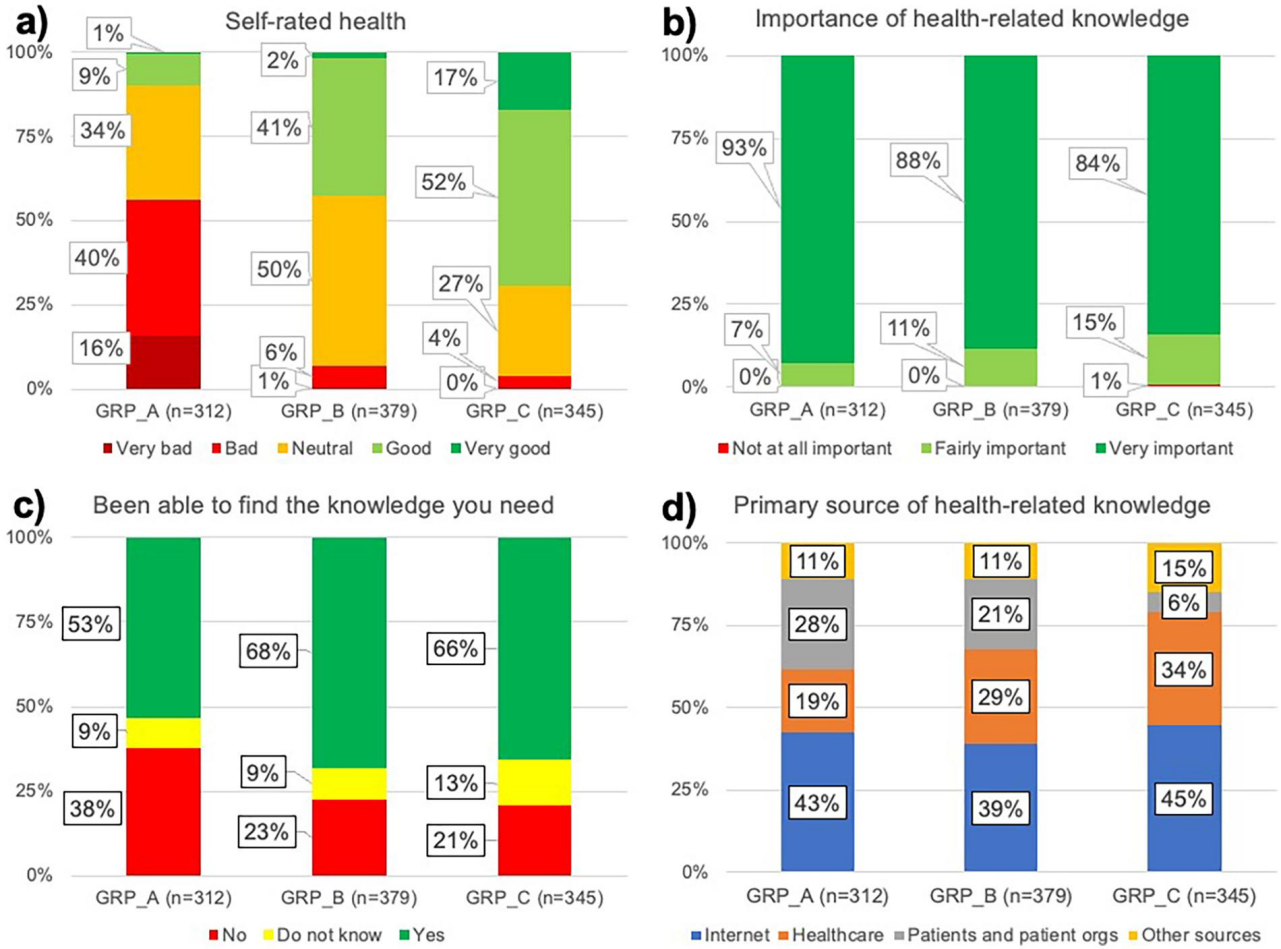
Visualization of survey responses from groups with different levels of disability for **(a)** self-rated health, **(b)** importance of health-related knowledge, **(c)** ability to find the knowledge they need, and **(d)** primary source of health-related knowledge.

Chi-square tests of independence showed that people living with high levels of disability were significantly more likely to report bad or very bad health (*Χ*^2^ (6, *N* = 1,036) = 484.12, *p* < .00001), to consider health-related knowledge important (*Χ*^2^ (4, *N* = 1,036) = 12.32, *p* = .015), to be less likely to have been able to find the health-related knowledge they need (*Χ*^2^ (4, *N* = 1,036) = 31.81, *p* < .00001), to be less likely to report healthcare as the primary source of health-related knowledge, and to be more likely to report other patients and patient organizations as their primary source of health-related knowledge (*Χ*^2^ (6, *N* = 1,036) = 62.00, *p* < .00001). See visualizations in Figures 2a–d.

### 3.2 Importance-performance analysis

**Figure 3:**
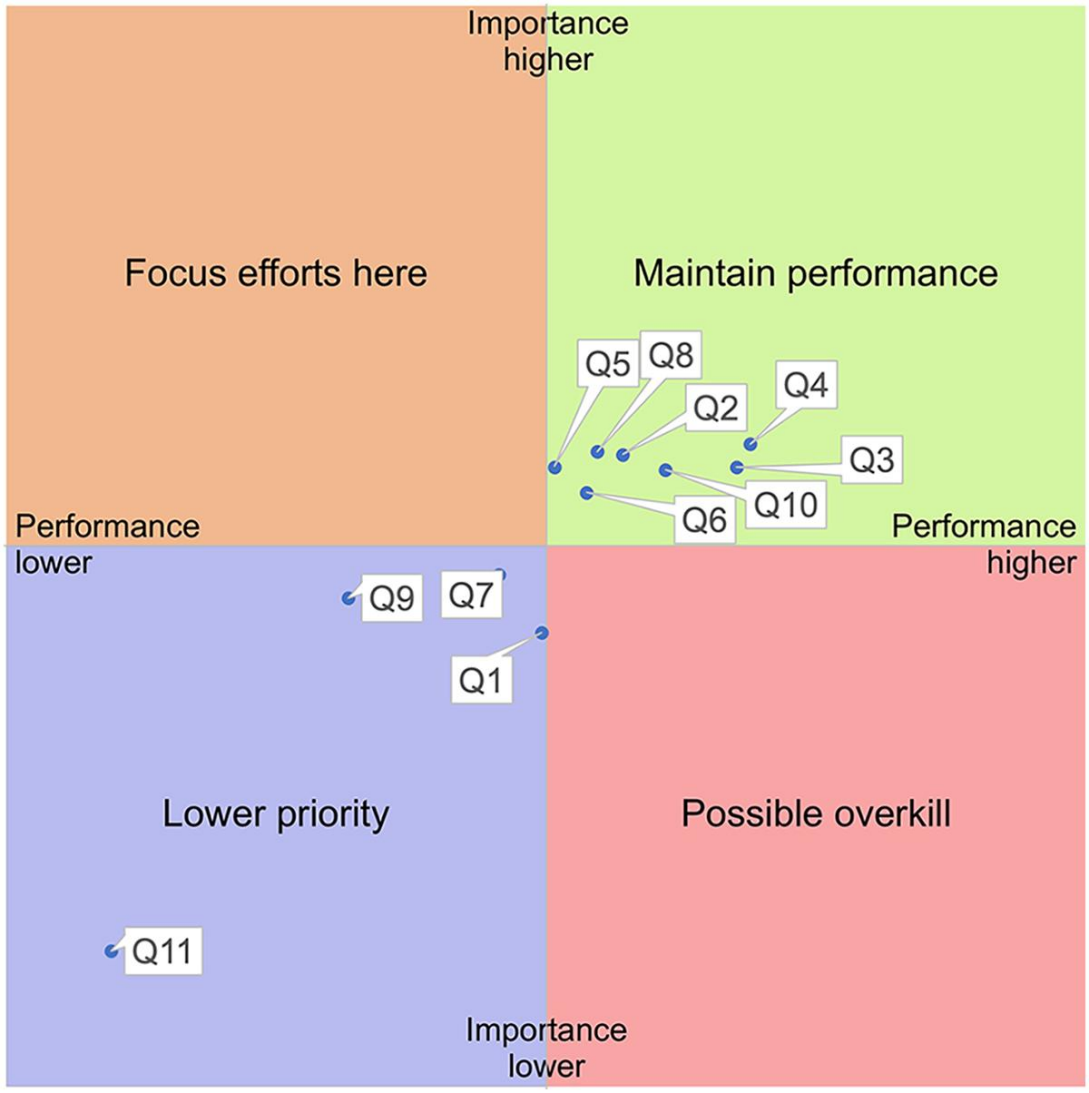
Results for all care dimensions (Q1-Q11).

The distribution of our different care dimensions in an Important-Performance grid can be seen in Figure 3. For each dimension (Q1-Q11), the mean of all responses for importance and performance respectively was calculated and placed in the grid. The point where the axes meet is the mean for all dimensions for importance (vertical axis) and performance (horizontal axis) respectively. See Table 3 for mean and median values.

**Table 3:** Mean and median values of importance and performance for all respondents for each care dimension (Q1-Q11) and total mean values.

Importance or Performance	Q1	Q2	Q3	Q4	Q5	Q6	Q7	Q8	Q9	Q10	Q11	Mean
Importance (mean)	4.61	4.92	4.90	4.94	4.90	4.86	4.71	4.93	4.67	4.90	4.04	4.76
Importance (median)	5	5	5	5	5	5	5	5	5	5	4	
Performance (mean)	3.64	3.78	3.98	4.01	3.66	3.72	3.56	3.74	3.30	3.86	2.88	3.65
Performance (median)	4	4	4	4	4	4	4	4	4	4	3	

The IPA for the full sample showed a clear distribution of our care dimensions into two relative groups: those where current performance should be maintained and those of lower priority (Figure 3). Most dimensions, including HCP's disease-specific knowledge (Q2), shared decision making (Q3), collaboration with HCP (Q4), effective and timely care (Q5), communication preferences (Q6), HCP responsiveness (Q8), and access to information (Q10), fell into the “maintain performance” quadrant, reflecting high importance and acceptable performance. In contrast, relational continuity (Q1), support for self-care (Q7), informational continuity (Q9), and co-design (Q11) clustered in the “lower priority” area, characterized by lower relative importance and lower performance. Importantly, these placements reflect *relative* rather than absolute priorities, and no dimension appeared in the “focus efforts here” quadrant for the overall sample.

We also analyzed the care dimensions for subgroups based on level of disability, which resulted in a different distribution of priorities. For six of the dimensions (Q1, Q4, Q6, Q8, Q10, Q11), individuals with high levels of disability rated importance higher than individuals with medium or no disability and for all 11 dimensions, individuals with high levels of disability rated performance lower than individuals with medium or no disability, see Table 4.

**Table 4:** Mean and median values of importance (IMP) and performance (PER) for each care dimension per level of disability.

Importance (IMP) or Performance (PER)	Level of disability	Q1	Q2	Q3	Q4	Q5	Q6	Q7	Q8	Q9	Q10	Q11
IMP (mean)	GRP_A	4.77	4.92	4.90	4.96	4.89	4.89	4.68	4.95	4.66	4.94	4.10
	GRP_B	4.65	4.95	4.94	4.94	4.92	4.84	4.71	4.93	4.68	4.89	4.06
	GRP_C	4.39	4.90	4.85	4.94	4.89	4.84	4.73	4.91	4.67	4.87	3.97
IMP (median)	GRP_A	5	5	5	5	5	5	5	5	5	5	4
	GRP_B	5	5	5	5	5	5	5	5	5	5	4
	GRP_C	5	5	5	5	5	5	5	5	5	5	4
PER (mean)	GRP_A	3.47	3.25	3.61	3.66	3.17	3.37	3.13	3.32	2.97	3.61	2.57
	GRP_B	3.67	3.89	4.03	4.07	3.78	3.82	3.65	3.82	3.41	3.96	2.93
	GRP_C	3.79	4.16	4.32	4.26	3.99	3.93	3.88	4.02	3.47	3.98	3.13
PER (median)	GRP_A	4	4	4	4	3	4	3	4	3	4	3
	GRP_B	4	4	4	4	4	4	4	4	4	4	3
	GRP_C	4	5	5	5	4	4	4	4	3	4	3

**Figure 4:**
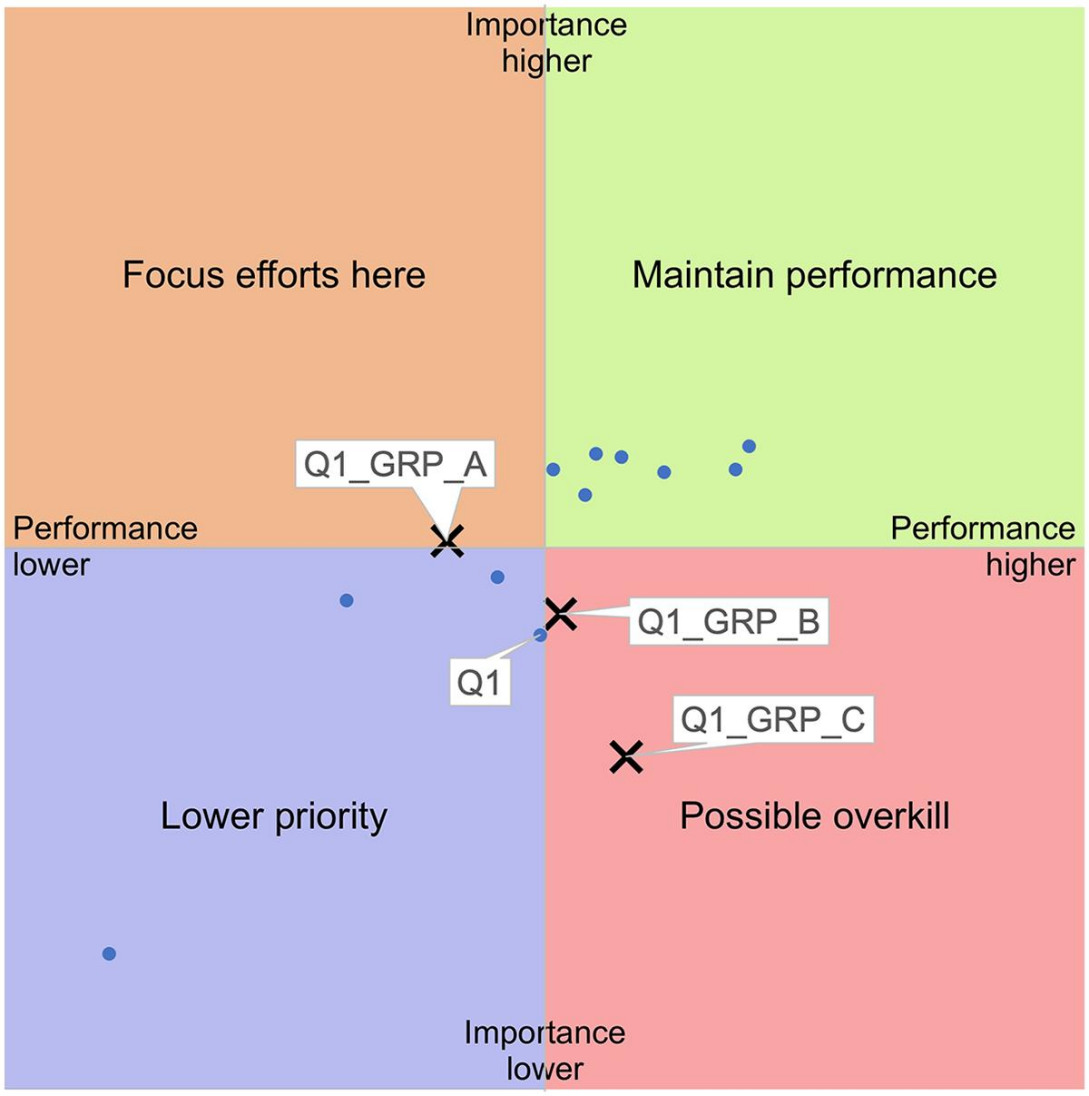
Results for Q1 (relational continuity) by level of disability (GRP_A: high, GRP_B: medium, GRP_C: none).

**Figure 5:**
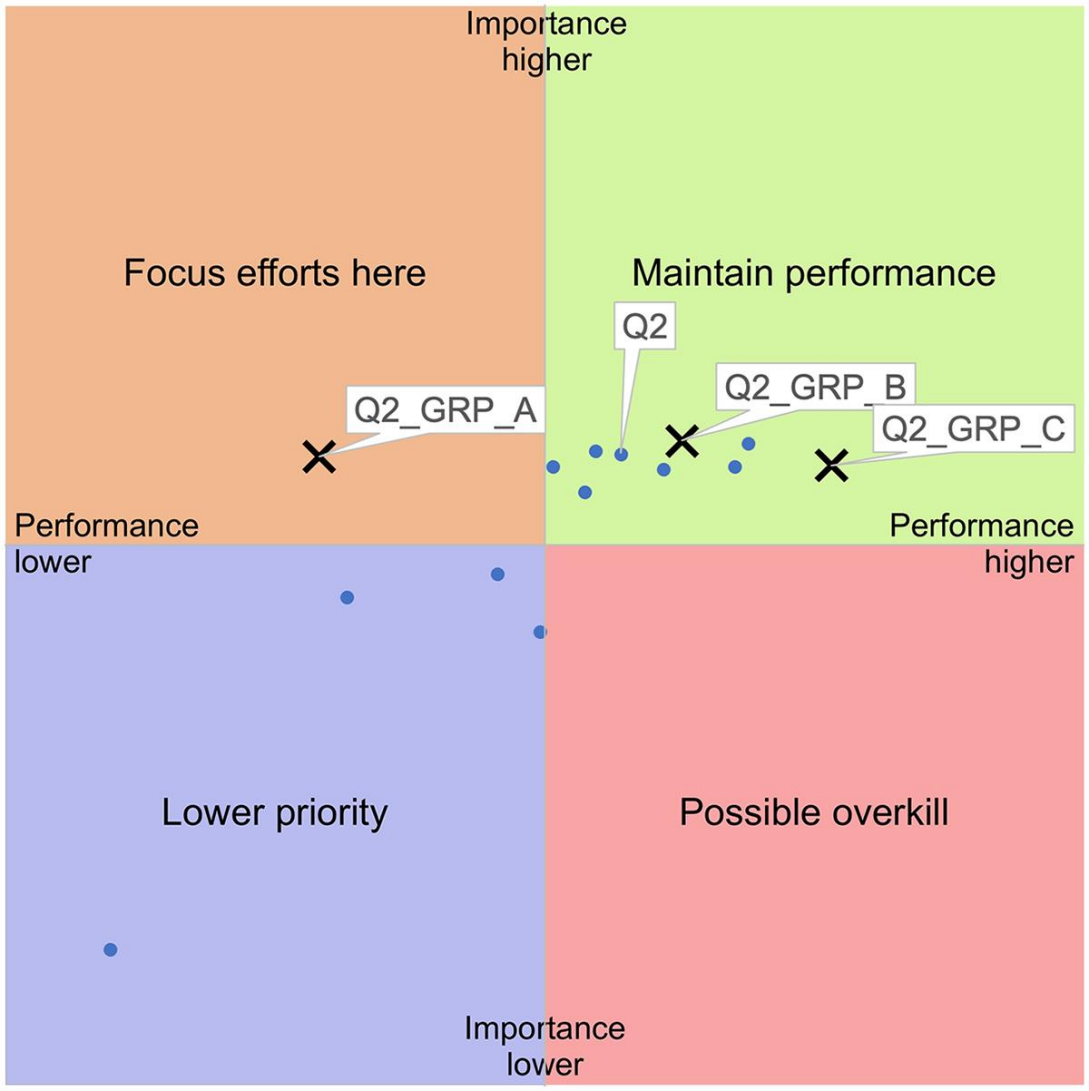
Results for Q2 (HCP's disease-specific knowledge) by level of disability (GRP_A: high, GRP_B: medium, GRP_C: none).

The largest difference in rated importance (vertical axis) between groups was found in relational continuity (Q1), see Figure 4, and for performance (horizontal axis) in HCP's disease-specific knowledge (Q2), see Figure 5.

Figure 4 shows clear subgroup differences for relational continuity (Q1). For respondents with high levels of disability (GRP_A), Q1 shifts into the “focus efforts here” quadrant, reflecting high importance and low performance. For respondents with medium levels (GRP_B) and no disability (GRP_C), Q1 appears in the quadrant “possible overkill,” suggesting lower perceived importance and higher performance. This gradient highlights relational continuity as an equity-relevant deficiency.

Figure 5 displays subgroup distributions for Q2 (HCPs’ level of knowledge). Although rated as important across all groups, perceived performance varies sharply: for respondents with high disability (GRP_A), Q2 is placed in the quadrant “focus efforts here,” indicating a substantial performance gap. In contrast, for GRP_B and GRP_C Q2 is placed in the “maintain performance” quadrant, with GRP_C reporting the highest perceived performance. This underscores significant disparities in how HCP are able to meet their patients’ needs depending on level of disability.

We received extensive comments in the free-text fields, these data were not analysed in the current study, however, those data will be used in a future manuscript.

## 4 Discussion

This study applied IPA to explore Swedish patients’ satisfaction with healthcare, meaning the alignment between what they value in healthcare and what they experience in practice on eleven different care dimensions. Our survey of 1,036 respondents revealed large and systematic differences between people with high, medium, and no disability, based on perceived activity limitation due to long-term health problems. Respondents in the high-disability group, consistently reported poorer health, greater difficulty accessing the knowledge they need, and were less likely to rely on healthcare as their primary information source. Instead, they turned more often to peers and patient organizations, suggesting substantial unmet informational needs in the healthcare system.

The IPA further highlighted these inequities. While the full sample showed two main clusters of care dimensions—those to maintain and those of lower priority—the subgroup analysis revealed that respondents with high levels of disability rated importance higher for most dimensions and rated performance lower for all dimensions. In particular, relational continuity (Q1, Figure 4) and healthcare professionals’ disease-specific knowledge (Q2, Figure 5) emerged as critical gaps for this group, falling into the quadrant “focus efforts here” when plotted separately. These findings point to structural shortcomings affecting those with the highest needs and suggest that improvement efforts should prioritize continuity, HCPs’ disease-specific knowledge, and informational support for people living with higher levels of disability.

Although descriptive surveys of patient experience are common, IPA adds value by generating actionable, priority-sensitive insights. Rather than reporting isolated satisfaction scores, IPA situates each care dimension within a relative priority structure, clarifying *where improvement efforts will matter most* and *for whom* (17). This is central to improvement science, which seeks not only to identify problems but to support meaningful and targeted change. Our findings therefore provide a clear starting point for quality improvement: maintain strong performance in shared decision making, communication, and responsiveness, while addressing the performance deficits in relational continuity, informational continuity, support for self-care, and co-design, particularly for patients with higher levels of disability.

### 4.1 Interpreting the key findings

In research, three concepts are commonly distinguished within continuity of care: relational continuity, organizational continuity, and informational continuity (27). Relational continuity (seeing the same HCP each visit) is often treated as the central aspect of patients’ and relatives’ perceptions of what matters most the most for them in healthcare (30). Informational continuity refers e.g., to integrated medical record-keeping and effective transfer of information. Organizational continuity encompasses coordination of care or treatment continuity and is achieved by ensuring that appropriate care is provided at the right time (27). High levels of continuity in healthcare have been shown to yield positive outcomes, and the importance of strong relational continuity has been widely reported (30). In our results, patients’ priorities and experiences showed substantial variation across disability groups, highlighting that “average” measures can mask important inequities. While relational continuity (27) was not a high priority overall, it was significantly more important to respondents with higher disability levels, who simultaneously reported the lowest performance. This suggests that continuity is not a universal need but a *conditional* one, and probably often especially relevant for patients with chronic and long-term conditions.

Similarily, disease-specific knowledge of HCP (Q2) showed the largest performance gap for the high-disability group, reflecting the particular importance of healthcare systems to meet the needs of patients with chronic and long-term conditions with HCPs equipped with sufficient levels of knowledge.

These findings refine rather than contradict prevailing person-centered care discourse. They indicate that responsiveness, respect, timely care, and effective communication remain consistently valued, but that continuity and HCP's disease-specific knowledge become critical for those with higher disability. This aligns with international evidence showing that people with disabilities report poorer care coordination, lower satisfaction, and more barriers despite universal healthcare (15, 16). Addressing these gaps should therefore be a central equity priority in Swedish healthcare.

Our results also align with IPA studies in England and Spain showing that timeliness, communication, and staff competence are strong drivers of patient experience (18, 19). However, the relatively low overall priority assigned to relational continuity contrasts with findings from some countries and challenges assumptions that continuity is universally important. Instead, our subgroup analysis suggests that continuity becomes a high-priority need specifically among patients with greater disability or complexity. This nuance is rarely examined in previous IPA studies and underscores the value of including equity-relevant variables in patient experience research.

The distinction between primary and secondary patient engagement helps interpret our findings. The care dimensions Q1-Q10 reflect primary engagement, meaning patients’ direct interactions with healthcare. Only Q11 concerns secondary engagement through participation in service development. Its placement in the “lower priority” quadrant suggests that many patients, especially those with lower levels of disability, do not prioritize contributing beyond their own care. However, respondents with high disability assigned slightly greater importance to co-design, reflecting their stronger need to influence system-level features that affect their daily lives. This supports earlier work showing that meaningful co-production often emerges from those with the most extensive contact with healthcare (31).

### 4.2 Implications for policy and practice

These findings have several implications for improving person-centered and equitable care. For patients with higher levels of disability, who often have more complex conditions, greater motivation to remain well, and higher informational needs, healthcare organizations should prioritize strengthening relational continuity and clinicians’ level of knowledge. Improving information transfer, both across providers and to patients, should be an explicit target for quality improvement, recognizing that patients today have rapidly expanding access to health information and that effective care increasingly relies on collaboration rather than the assumption that clinicians can “know everything.”

IPA offers a practical decision-support tool for identifying where action is most needed. By visualizing discrepancies between what patients value and what they experience, IPA highlights priority-sensitive gaps and equity issues that remain hidden in aggregate metrics. Although IPA cannot explain underlying causes, its strength lies in integrating patient priorities into iterative improvement cycles. Used within PDSA frameworks, IPA can guide the identification of baseline gaps, support targeted interventions such as communication training or coordination enhancements, and enable follow-up assessments to determine whether changes improve patient experience (20).

Practically, healthcare teams can use IPA results to strengthen communication, responsiveness, and coordination along patient journeys, and to anchor structured dialogues with patients about local improvement priorities. Importantly, disability should be treated as a segmentation variable in quality measurement to avoid masking inequities in average scores. Taken together, these implications position IPA as a complementary method within improvement science—helping organizations move from measurement to meaningful, patient-informed change.

A key principle of improvement science is to move beyond describing problems toward implementing and studying change (1). The IPA approach directly supports this principle by identifying where action is most needed. Because it produces intuitive visualizations of discrepancies between importance and performance (17–19), IPA can be used to guide local quality improvement teams in setting priorities that are meaningful to patients.

### 4.3 Methodological reflections

The study has both strengths and limitations. One of the main strengths is that IPA offers a straightforward way to integrate patient voice into quality measurement by translating complex survey data into actionable priorities. Nevertheless, certain limitations should be noted. The sample was geographically diverse and covered a broad range of background and disability levels. We did however have an over-representation of women, people older than 50, and people with tertiary education level, which affects how our results can be interpreted. In addition, while IPA effectively highlights gaps between patient priorities and experiences, it does not identify underlying causes or prescribe specific solutions. The analysis relies on subjective perceptions, which may shift over time, and requires local interpretation to guide actionable change.

The convenience sampling strategy and the limitations mentioned above means the results cannot be interpreted as representative for the population as a whole; it is likely that more engaged or health-literate individuals participated, leading to limitations in inclusiveness and equity. However, for this observational study our chosen strategy allowed a quick data collection and to inform future studies where statistical representativeness can be sought.

The IPA analysis could result in validity problems since it does not adequately address potential sources of bias (32), and overall the four-quadrant matrix could oversimplify the complexity of importance and performance. Furthermore, disability was self-reported and captures perceived activity limitation; subgroup boundaries may therefore be heterogeneous. Self-reported data are subject to recall bias, and cross-sectional design precludes conclusions about causality or change over time. In larger samples, further refinement (e.g., separating “no long-term problem” from “long-term problem without limitation”, or stratifying by type of limitation) would enable more granular subgroup analyses.

### 4.4 Future perspectives

The IPA approach can be embedded in healthcare governance structures as part of a continuous Learning Health System (5, 6). A practical model could include four steps: first, measure patient priorities and experiences to establish a baseline; second, identify discrepancies and select focus areas collaboratively; third, implement targeted improvements; and fourth, re-measure to assess progress. This cycle could be linked to national and regional efforts to ensure sustained organizational learning. Such integration would allow healthcare providers and policymakers to align improvement efforts with what patients actually value and to monitor equity impacts across groups. Future studies could use stratified random sampling, combine IPA with qualitative interviews, or integration into longitudinal improvement programs to better capture the dynamics of change.

## 5 Conclusions

Our findings demonstrate that IPA can be an effective tool for both evaluating and guiding quality improvement initiatives, and that patient priorities vary across groups, especially by disability level. By identifying areas where performance falls short of importance, we aimed to provide actionable insights for improving healthcare quality from the patient perspective.

These findings suggest that, from the patient perspective, Swedish healthcare could improve its responsiveness and adaptability. Taken together, our findings indicate that IPA is not only an analytical framework but also a practical tool for guiding and evaluating patient-centered improvement. By illuminating where importance and performance diverge, and for whom, IPA helps ensure that improvement efforts address what matters most to patients. Applying this approach systematically across Swedish healthcare could strengthen the pursuit of equity, person-centeredness, and continuous learning.

## Data Availability

The raw data supporting the conclusions of this article will be made available by the authors, without undue reservation.
