# Supplementary material for: Making patient experience actionable: applying importance-performance analysis to guide improvements in Swedish healthcare
Source: Front Health Serv. 2026 Mar 17;6:1765906. doi: 10.3389/frhs.2026.1765906 (PMC13036185; doi:10.3389/frhs.2026.1765906)
Supplement: Supplementary file 1 [file Datasheet1.pdf]

Supplementary Material

1 Survey Questions

1.1 Background

- Gender identity
    - female/male/other/do not want to respond
- Age
    - 18-29/30-49/50-69/70-
- What is your highest completed level of education?
    - Primary/secondary/tertiary
- Which region do you live in?
    - Dropdown with all regions in Sweden listed
- Current employment status
    - Employed/self-employed/unemployed/old age pensioner/on parental leave/on disability/other
- Do you have a long-term illness, injury-related problems, or any other long-term health condition?
    - Yes/no
- If yes to the previous question – Do these problems reduce your ability to work or hinder you in your other daily activities?
    - Yes, to a large degree/yes, to some degree/no, not at all
- Are you a relative/caregiver of someone with a long-term illness or health condition?
    - Yes/no
- How would you assess your general health?
    - Very good/good/neutral/bad/very bad
- How important is it for you to have knowledge about your health and health challenges?
    - Very important/fairly important/not important
- Have you been able to acquire the knowledge you need about your health and healthcare?
    - Yes/no/don't know
- What is your main source of knowledge about health and healthcare?
    - Healthcare/family or friends/found myself online/other patients/patient organizations/other sources
- Where did you receive care on your most recent healthcare occasion?
    - Primary care/secondary care/emergency care/other
- Was your most recent healthcare visit digital or in-person?
    - Digital/in-person

1.2 Care dimensions

Response options for all were a 5-point Likert scale: 1 = strongly disagree, 3 = neutral, 5 = strongly agree

- Q1 Importance: It is important to me to repeatedly meet the same healthcare staff during visits (e.g., doctor).
- Q1 Performance: My most recent healthcare visit fulfilled this for me.
- Q2 Importance: It is important to me that the healthcare staff are knowledgeable about the condition(s)/problem(s) I have.
- Q2 Performance: My most recent healthcare visit fulfilled this for me.
- Q3 Importance: It is important to me that the healthcare staff involve me in decisions regarding my care.
- Q3 Performance: My most recent healthcare visit fulfilled this for me.
- Q4 Importance: It is important to me to have good collaboration with the healthcare staff.
- Q4 Performance: My most recent healthcare visit fulfilled this for me.
- Q5 Importance: It is important to me that I receive the right kind of care without unnecessary waiting.
- Q5 Performance: My most recent healthcare visit fulfilled this for me.
- Q6 Importance: It is important to me to be able to communicate with healthcare in the ways that suit me best — during visits, before visits, between visits, etc.
- Q6 Performance: My most recent healthcare visit fulfilled this for me.
- Q7 Importance: It is important to me that the healthcare staff support me in selfcare, that is, support me in taking care of my own health as much as possible.
- Q7 Performance: My most recent healthcare visit fulfilled this for me.
- Q8 Importance: It is important to me that the healthcare staff take in and consider the information I want to share.
- Q8 Performance: My most recent healthcare visit fulfilled this for me.
- Q9 Importance: It is important to me that different care providers have access to the same information about me.
- Q9 Performance: The healthcare system has fulfilled this for me.
- Q10 Importance: It is important to me that I have access to the information that the healthcare system has about me (such as my record, test results, and similar).
- Q10 Performance: The healthcare system has fulfilled this for me.
- Q11 Importance: It is important to me to have the opportunity to participate in the development and organization of healthcare beyond my own appointment, for example via patient or user advisory councils.
- Q11 Performance: The healthcare system has fulfilled this for me.
